# Induction of connective tissue growth factor accounts for the inability of glucocorticoid suppression on pulmonary fibrosis

**DOI:** 10.1002/ctm2.867

**Published:** 2022-05-27

**Authors:** Zhaoni Wang, Xiangsheng Yang, Xin Xu, Qingyang Yu, Yang Peng, Jianxing He, Nanshan Zhong, Xiao Xiao Tang

**Affiliations:** ^1^ State Key Laboratory of Respiratory Disease National Clinical Research Center for Respiratory Disease National Center for Respiratory Medicine Guangzhou Institute of Respiratory Health The First Affiliated Hospital of Guangzhou Medical University Guangzhou China; ^2^ Guangzhou Laboratory, Bio‐island Guangzhou Guangdong China

Dear Editor,

Glucocorticoid (GC) therapy works in some types of interstitial pneumonia but has an unfavourable efficacy in idiopathic pulmonary fibrosis (IPF).^1^, ^2^ The underlying mechanism remains elusive. Here, we determined that the inability of GC suppression on pulmonary fibrosis is not attributed to impairment of steroid sensitivity, but due to induction of connective tissue growth factor (CTGF), which promotes fibronectin expression and fibroblast‐to‐myofibroblast differentiation.

The expression of GC receptor (GR, encoded by *Nr3c*) was positively correlated to GC sensitivity. Previous studies reported that GRα expression was downregulated in IPF lungs as compared to those in steroid‐sensitive interstitial lung diseases (ILDs),^3^, ^4^ thus proposing that the inefficiency of GC therapy in IPF might be ascribed to decreased steroid sensitivity. However, this is debatable. What's more, previous studies lacked dynamic evaluation on steroid responsiveness. In this study, we performed more measurements on GC sensitivity and responsiveness in samples of both human subjects and mouse model. Other than GR, histone deacetylase 2 (HDAC2) and 11β‐hydroxysteroid dehydrogenase type 1 (HSD11b1) are also essential for GCs to take effect.^5^ With greater sample size, we demonstrated that expressions of *GRα*, *HDAC2* and *HSD11b1* in lung tissues were comparable between healthy subjects and IPF patients (Figure 1A). In mice with bleomycin‐induced lung fibrosis (BLM mice), similar results were observed (Figure 1B). Although *Hsd11b1* expression was downregulated after the BLM treatment, it returned to the normal level upon Dexamethasone (DEX) stimulation (Figure 1B). We also detected the dynamic responses of IPF lung cells and BLM mice to the GC treatment. DEX increased nuclear expression of GRα in human lung fibroblasts (hLFs) derived from healthy controls and IPF patients (Figure 1C, Figure S1), indicating formation of the GC‐GRα complex, which regulates transcription of the target genes in cell nucleus.^5^ GC‐inducible genes were also upregulated upon the DEX treatment in hLFs (Figure 1D, Figure S2), human airway epithelial cells (Figure S3) and BLM mice (Figure 1E). And the expressions of GC‐inducible genes between HC and IPF were not markedly different. Since GCs have an acknowledged anti‐inflammatory effect, we assessed lung inflammation in BLM mice and found that BLM‐induced production of inflammatory cytokines could be suppressed following the DEX treatment (Figure 1F). These findings suggested that the steroid sensitivity and responsiveness in IPF cells and BLM mice are not impaired.

**FIGURE 1:**
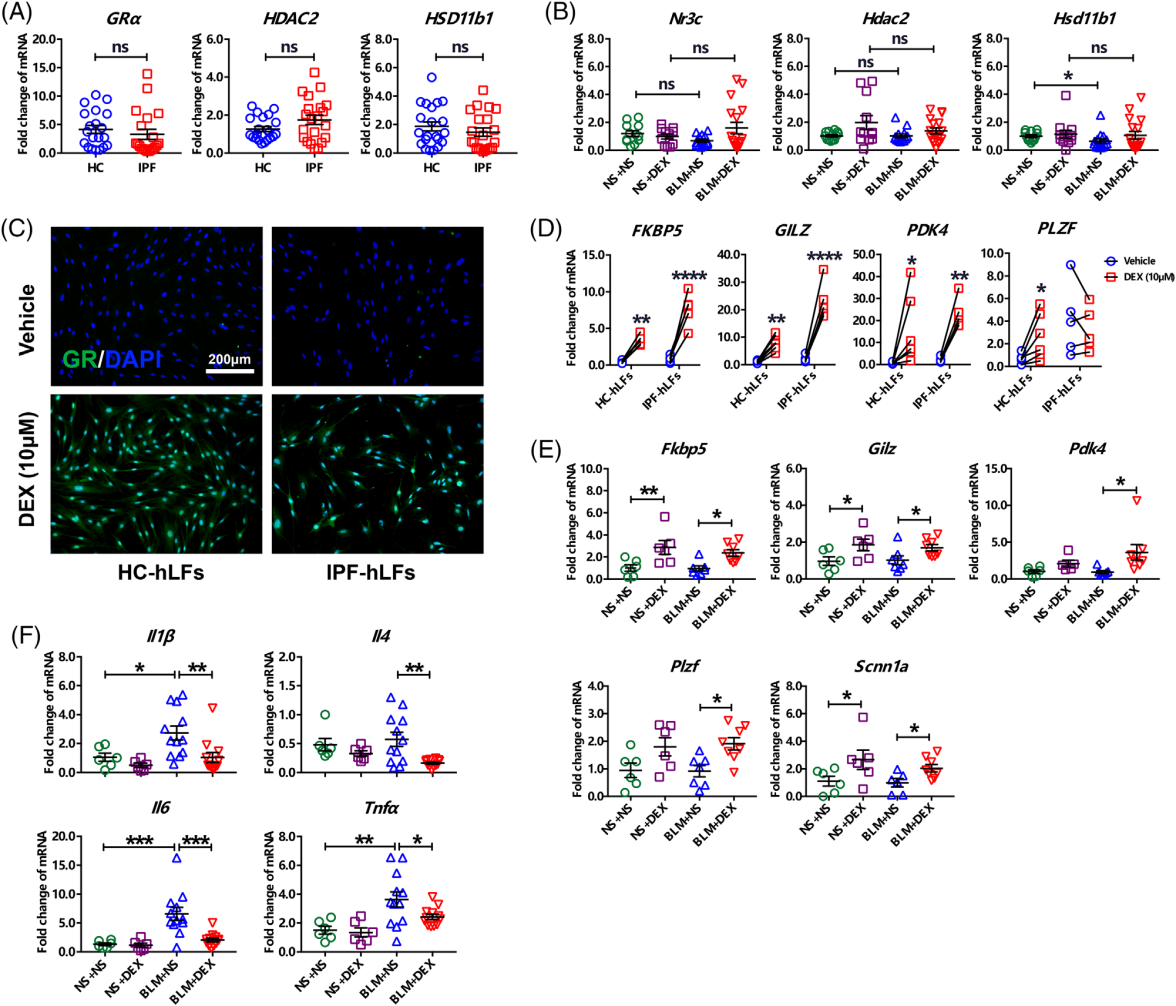
Lung tissue from idiopathic pulmonary fibrosis (IPF) patients and bleomycin (BLM)‐treated mice is responsive to glucocorticoid treatment. (A) Transcriptional expressions of glucocorticoid sensitivity‐related genes in the lung tissues of IPF patients vs. healthy controls (HC) (*n* = 20 and 20, respectively). (B) Transcriptional expressions of glucocorticoid sensitivity‐related genes in the mouse lung tissue (*n* = 12, 11, 13 and 17, respectively). NS, normal saline; DEX, dexamethasone; “BLM/NS + DEX/NS”, mice were intratracheally administrated with bleomycin or normal saline on the first day, and then received DEX or NS injection every day of the following three weeks. (C) The nuclear expression of GRα in human lung fibroblasts (hLFs) following 2 h of DEX stimulation. (D,E) Expression changes of the glucocorticoid‐inducible genes following DEX stimulation in hLFs (D, *n* = 5 and 5, respectively) and mouse lung (E, *n* = 6, 6, 7 and 8, respectively). (F) Perturbance of cytokine levels in the mouse lungs upon BLM treatment and DEX therapy (*n* = 6, 6, 12 and 12, respectively). Unpaired variables are expressed as means ± SEM. *, **, ***, **** or ns (no significance) represent *P* value less than 0.05, 0.01, 0.001, 0.0001 or larger than 0.05, respectively. FKBP5, FK506 binding protein 5. GILZ, glucocorticoid‐induced leucine zipper. PDK4, pyruvate dehydrogenase kinase 4. PLZF, promyelocytic leukaemia zinc finger. SCNN1A, sodium channel epithelial 1 subunit alpha

Bleomycin‐induced inflammation is an important contributor to lung fibrosis in mice. However, early anti‐inflammatory intervention with DEX did not attenuate the pathology of lung fibrosis (Figure 2A,B, Figure S4A) or collagen deposition (Figure 2A,C) in BLM mice. DEX also had no improvement in survival or body weight of the mice (Figure S4B, S4C). In parallel, DEX had minimal effect on BLM‐induced overexpression of fibrosis‐related factors, including fibronectin, type I and III collagens as well as lysyl oxidase (Lox) (Figure 2D, Figure S4D), and even further upregulated α‐smooth muscle actin (α‐SMA) (Figure 2D), which indicates transdifferentiation of fibroblasts into myofibroblasts. In IPF‐derived hLFs, DEX enhanced α‐SMA and fibronectin expression (Figure 2E,F), in a concentration‐dependent manner from 0.01 μM to 10 μM (Figure S5).

**FIGURE 2:**
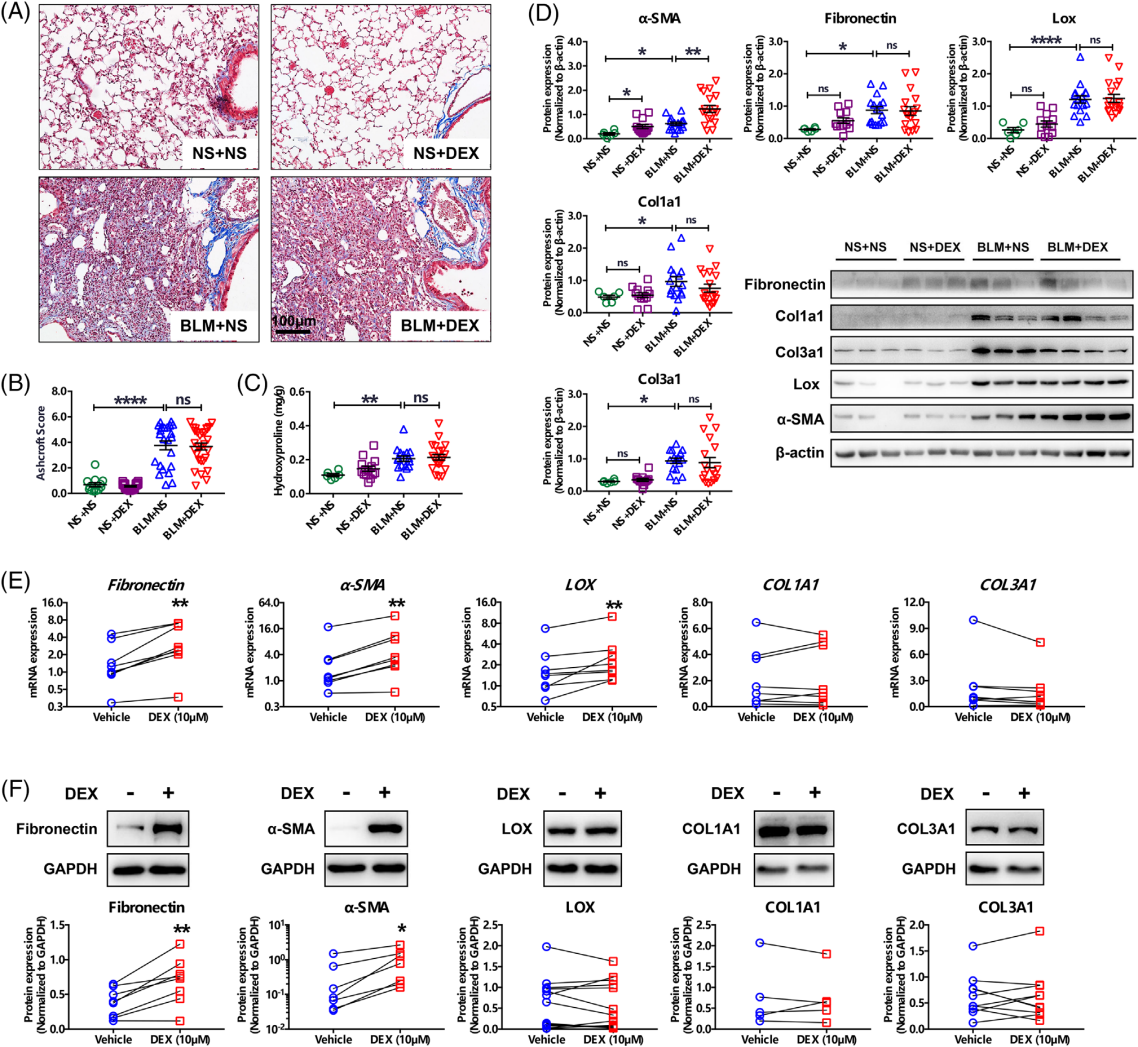
Glucocorticoid fails to ameliorate bleomycin (BLM)‐induced pulmonary fibrosis in mice, and promotes fibronectin and α‐smooth muscle actin (α‐SMA) expression in idiopathic pulmonary fibrosis‐human lung fibroblasts (IPF‐hLFs). (A) Masson staining of the mouse lung sections, with blue staining indicating collagen deposition. (B) Ashcroft scoring of lung histopathology (*n* = 12, 20, 23 and 29, respectively). (C) Hydroxyproline content of mouse lungs (*n* = 6, 14, 16 and 21, respectively). (D) Protein levels of the fibrosis‐related factors in mouse lung tissues (*n* = 6, 12, 16 and 18, respectively). (E) Transcriptional expression of fibrosis‐related factors in IPF‐derived hLFs following DEX treatment (*n* = 8). (F) Protein expression of fibrosis‐related factors in IPF‐derived hLFs following DEX treatment (*n* = 5–10). Data in (B)–(D) are expressed as means ± SEM. *, **, ***, **** or ns (no significance) represent *P* value less than 0.05, 0.01, 0.001, 0.0001 or larger than 0.05, respectively. “BLM/NS + DEX/NS”, mice were intratracheally administrated with BLM or normal saline (NS) on the first day, and then received dexamethasone (DEX) or normal saline injection daily for three weeks. α‐SMA, α‐smooth muscle actin. COL1A1/Col1a1, collagen type I alpha 1 chain. COL3A1/Col3a1, collagen type III alpha 1 chain. LOX/Lox, lysyl oxidase

It was previously reported that low‐dose and early short‐course of the GC treatment could better attenuate BLM‐induced lung fibrosis in rats than the high‐dose or long‐course strategy did,^6^ suggesting that the inability of GC suppression on lung fibrosis was not correlated to insufficient dosage or treatment course. Our study also indicated a positive dose–response relationship between GC response and DEX treatment in IPF‐hLFs (Figure S2), while this response seems to lead to pro‐fibrosis instead of anti‐fibrosis. To explore the mechanism underlying induction of α‐SMA and fibronectin by DEX, we detected several profibrotic factors in mouse lung and IPF‐hLFs. In the lung of BLM mice, DEX reduced the expression of platelet derived growth factor‐b (*Pdgf‐b*) and transforming growth factor‐β1 (*Tgf‐β1*), whereas increased *Ctgf* level (Figure 3A). Consistently, DEX also downregulated *TGF‐β1* while upregulated CTGF expression in IPF‐hLFs (Figure 3B,C), implicating the critical role of CTGF in mediating profibrotic effect of the DEX treatment.

**FIGURE 3:**
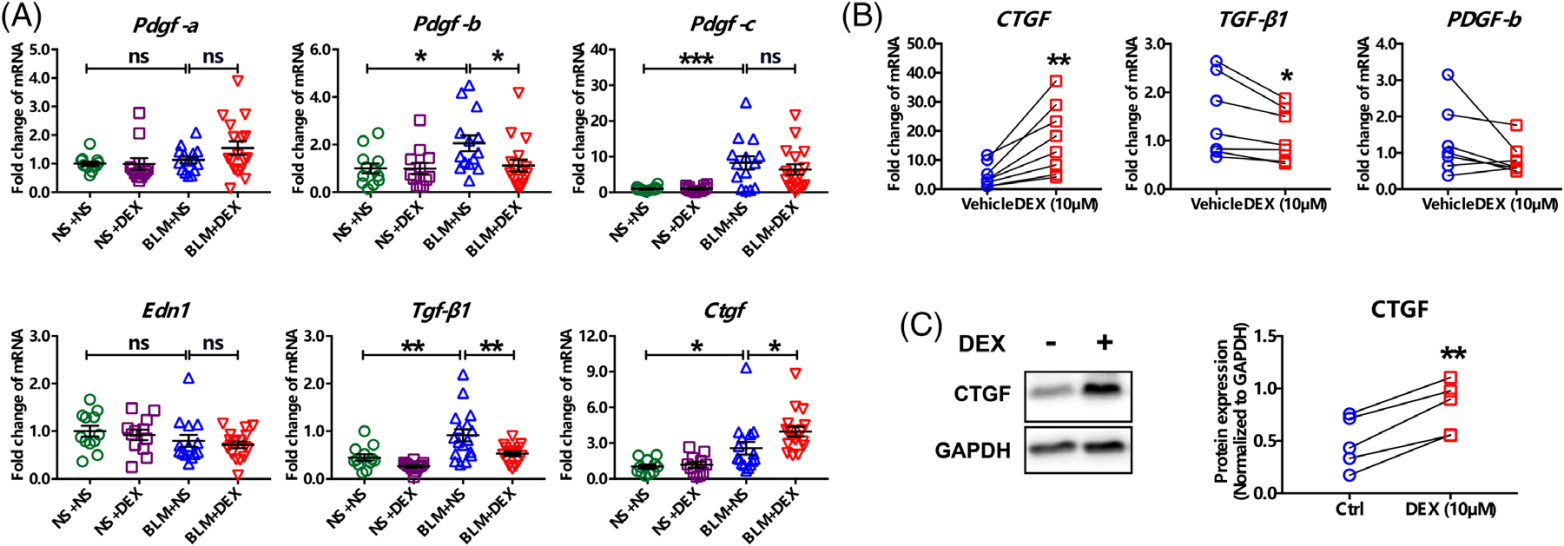
Dexamethasone (DEX) treatment enhances connective tissue growth factor (CTGF) expression in lung tissue of bleomycin (BLM)‐induced mice and IPF‐hLFs. (A) Expression change of profibrotic factors in mouse lung tissue upon BLM and DEX treatment (*n* = 12, 12, 14 and 17, respectively). Data are expressed as means ± SEM. (B) Transcriptional expression of TGF‐β1, PDGF‐b and CTGF in IPF‐derived hLFs following DEX treatment (*n* = 7). (C) Protein expression of CTGF in IPF‐hLFs upon DEX stimulation (*n* = 5). *, **, *** or ns (no significance) represent *P* value less than 0.05, 0.01, 0.001 or larger than 0.05, respectively. CTGF/Ctgf, connective tissue growth factor. Edn1, endothelin 1. PDGF/Pdgf, platelet derived growth factor. TGF‐β1/Tgf‐β1, transforming growth factor‐β1

Served as a downstream effector of TGF‐β, CTGF exerts a vital role in fibrogenesis.^7^ Our data also confirmed its elevation in IPF lung tissues (Figure S6). Phase II trial showed that CTGF monoclonal antibody (Pamrevlumab) slowed down lung function decline in IPF patients.^8^ Currently, phase III clinical trials (NCT03955146, NCT04419558) of Pamrevlumab for IPF patients are under way. To ascertain the role of DEX‐induced CTGF in lung fibrosis, we used a monoclonal antibody to neutralize the released CTGF, and found that DEX‐induced upregulation of α‐SMA and fibronectin was alleviated following neutralization (Figure 4A). Similarly, knockdown of CTGF using specific siRNA also reduced the production of fibronectin upon DEX treatment (Figure 4B). A previous study demonstrated that Caffeine can ameliorate CTGF expression induced by GCs in a human lung fibroblast cell line.^9^ We ascertained that Caffeine at 5 mM depressed DEX‐induced CTGF expression (Figure 4C). Although Caffeine has minor effect on fibronectin expression (Figure 4C), a positive correlation between fibronectin and CTGF protein level (Figure 4D) suggested that higher dose of caffeine would be effective. Notably, CTGF antibody and caffeine also remarkably decreased the expression of α‐SMA, collagen (I, III) and LOX (Figure 4A,C).

**FIGURE 4:**
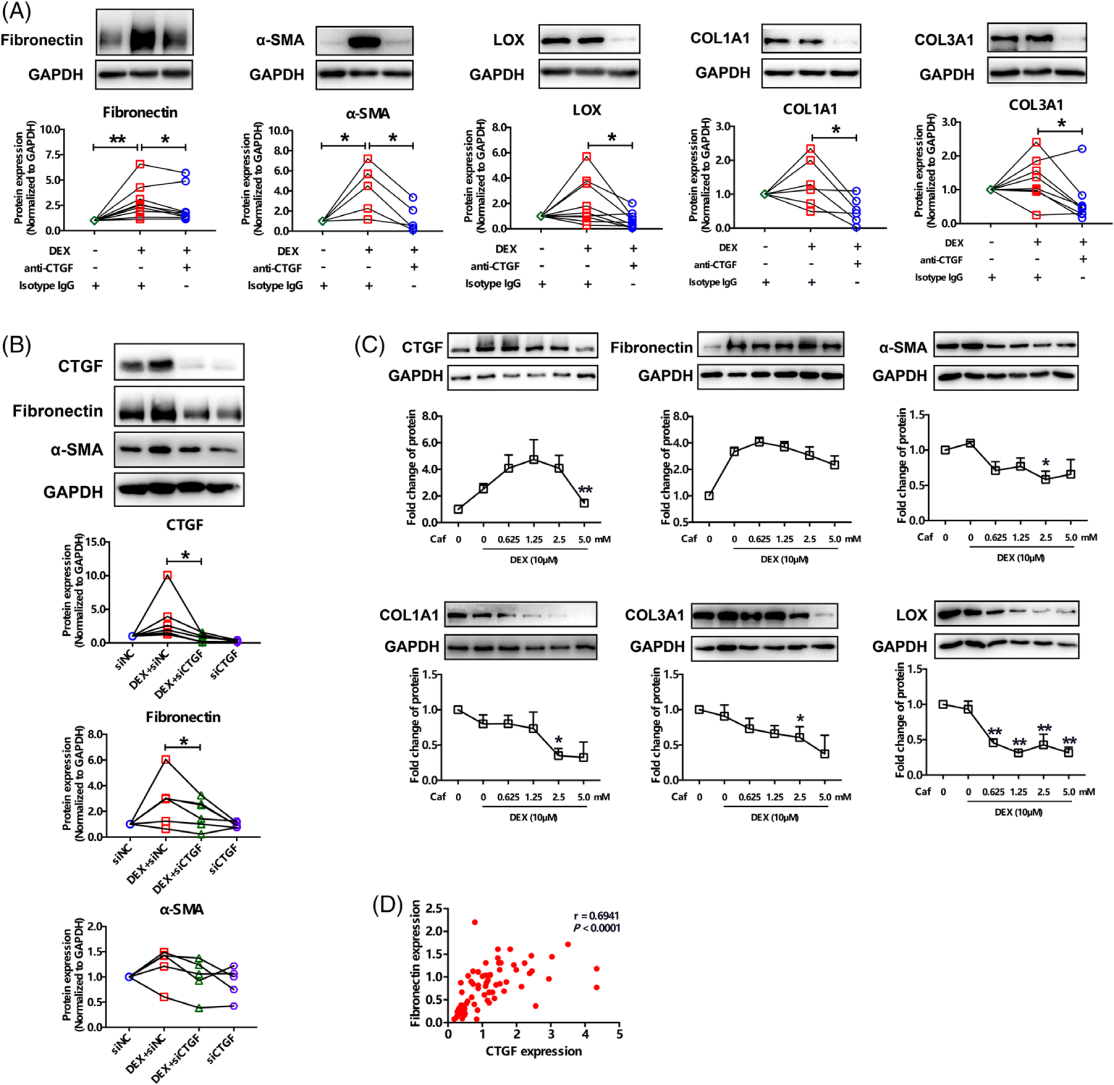
Upregulation of connective tissue growth factor (CTGF) accounts for dexamethasone (DEX)‐induced expression of fibrosis‐related factors. (A) Protein expression of fibrosis‐related factors in DEX‐treated IPF‐hLFs after neutralizing CTGF (*n* = 5–8). (B) Protein expression of CTGF and fibrosis‐related factors in DEX‐treated IPF‐hLFs after knockdown of CTGF through siRNA (*n* = 5–6). NC, non‐target control. siNC, NC siRNA. siCTGF, CTGF siRNA. (C) Protein expression of CTGF and fibrosis‐related factors in IPF‐hLFs treated by DEX (10 μM) with or without caffeine (0.625–5.0 mM) (*n* = 6–8). * and ** represent *P* < 0.05 and *P* < 0.01 when compared to hLFs treated with DEX and 0 mM Caf (the second group). Caf, caffeine. (D) Correlation between the protein expression of fibronectin and CTGF was analyzed by Pearson's correlation test. *, *P *< 0.05. ** *P *< 0.01

In summary, our study reveals the mechanism by which the GC treatment fails to suppress pulmonary fibrosis. DEX may exhibit “anti‐fibrotic” efficacy by downregulating TGF‐β1 and PDGF‐b, and also have “pro‐fibrotic” role by promoting CTGF expression. In vivo, these dual effects may be equivalent and thus lead to no change in lung fibrosis after DEX therapy. In vitro, the “pro‐fibrotic” force may be advanced over the other one, hence GC treatment facilitates expression of fibrotic factors. In breast cancer, DEX was reported to enhance CTGF expression through the PI3K‐SGK1 pathway,^10^ indicating a potential target for modulating GC‐induced CTGF in pulmonary fibrotic diseases, while this requires further investigation.

## CONFLICT OF INTEREST

The authors declare that there is no conflict of interest that could be perceived as prejudicing the impartiality of the research reported.

## FUNDING

The National High‐Level Talents Program (X.X.T.); The National Natural Science Foundation of China (81770015, X.X.T.); Local Innovative and Research Teams Project of Guangdong Pearl River Talents Program (2017BT01S155); Open Project of State Key Laboratory of Respiratory Disease (SKLRD‐OP‐202109); Special Fund for Science and Technology Innovation of Guangdong Province (2020B1111330001)

## AUTHORS' CONTRIBUTIONS

Xiao Xiao Tang and Nanshan Zhong conceived the study, Xiao Xiao Tang designed and supervised the study. Zhaoni Wang, Xiangsheng Yang, and Qingyang Yu conducted experiments. Xin Xu, Yang Peng, and Jianxing He contributed to clinical specimen collection. Xiao Xiao Tang and Zhaoni Wang interpreted the data and wrote the manuscript. All authors have read and approved the manuscript.

## Supporting information

Supporting Information
