# Supplementary material for: Induction of connective tissue growth factor accounts for the inability of glucocorticoid suppression on pulmonary fibrosis
Source: Clin Transl Med. 2022 May 27;12(5):e867. doi: 10.1002/ctm2.867 (PMC9136703; doi:10.1002/ctm2.867)
Supplement: Supplementary file 1 — Supporting Information [file CTM2-12-e867-s001.pdf]

SUPPLEMENTAL MATERIALS

Induction of connective tissue growth factor accounts for the inability of glucocorticoid suppression on pulmonary fibrosis

Zhaoni Wang, Xiangsheng Yang, Xin Xu, Qingyang Yu, Yang Peng, Jianxing He, Nanshan Zhong*, Xiao Xiao Tang*.

DETAILED METHODS

Human lung tissue collection

Collection of human lung tissue was approved by the Institutional Review Board of the First Affiliated Hospital of Guangzhou Medical University, and all subjects have provided written informed consent. IPF patients were included in this study according to the diagnostic criteria of 2018 ATS/ERS/JRS/ALAT Clinical Practice Guideline [E1]. Tissue from explanted IPF lungs or healthy donors was collected for isolation of lung fibroblasts and/or RNA extraction.

Human lung fibroblasts and treatment

Human lung tissues were cut into pieces and digested to isolate human lung fibroblasts (hLFs). Fibroblasts were grown in Dulbecco's modified eagle medium (DMEM, GIBCO) supplemented with 10% fetal bovine serum (Genetimes, Shanghai, China) and 1% antimycotic-antibiotic (GIBCO) at 37°C with 5% CO_2. Cells between passage 5 and 9 were used for experiments.

hLFs were treated with dexamethasone (DEX; Sigma) or equal volume of 1× dPBS in DMEM with 5% FBS for 4 or 48 hours. For immunofluorescence staining of glucocorticoid receptor, hLFs were serum-starved for 48 hours and then treated with DEX in serum-free DMEM for 2 hours. Rabbit anti-connective tissue growth factor (CTGF) antibody (1.25 μg/ml; Peprotech) was used to neutralize CTGF released by hLFs during DEX stimulation, with rabbit IgG (1.25 μg/ml; Yeasen, Shanghai, China) as isotype control. The sequence for CTGF-siRNA (RiboBio, Guangzhou, China) was GCACCAGCATGAAGACATA. 24 hours before DEX stimulation, 50 nM of

CTGF-siRNA or nontarget control (NC)-siRNA was transfected to hLFs. Caffeine (Caf, 0.625-5.0 mM, GLPBIO, CA, USA) was used combinedly with DEX in hLFs.

Animal model

8-week-old male C57bl/6 mice were purchased from Beijing HFK Bioscience Company (Beijing, China). Mice were housed in specific pathogen free facilities and maintained in standard cages with sterile food and water available *ad libitum*. All animal experimental procedures were approved by the Animal Care and Use Committee of Guangzhou Medical University (Approved number: 2021351), and confirmed to the Guide for the Care and Use of Laboratory Animals.

Mice were anesthetized with 0.25% avertin (25 mg/kg of body weight; Sigma). Bleomycin (BLM; Hanhui Pharma., Shanghai, China) was dissolved in 0.9% sterile normal saline (NS). Each mouse was intratracheally administrated with 2 mg/kg of BLM or equal volume of 0.9% NS. DEX was diluted with 0.9% NS to the concentration of 0.1 mg/ml. Since the next day after BLM exposure, 10 μl of DEX solution or 0.9% NS per gram of body weight was injected intraperitoneally to each mouse daily until sacrifice. According to whether receiving BLM stimulation and/or DEX treatment, mice were divided into 4 groups, referred to as "NS + NS", "NS + DEX", "BLM + NS" and "BLM + DEX".

Three weeks after BLM exposure, mice were over-anesthetized to death. The right lung lobe of each mouse were removed and frozen in liquid nitrogen immediately. The frozen lung tissue was ground into powder, aliquoted and stored in -80 ºC for RNA and protein extraction as well as hydroxyproline measurement. The left lung

lobe was perfused and fixed with 4% paraformaldehyde for further histological analysis. Specifically, for detecting the expression of inflammatory cytokines in lung tissues, mice were sacrificed one week after bleomycin instillation. For the measurement of glucocorticoid-inducible genes, lung tissues were collected within 4-6 hours after the final injection of dexamethasone.

RNA extraction and Real-Time quantitative PCR

The lung tissue RNA was extracted by using Nuclezol RNA Isolation Plus Reagent (Macherey-Nagel, Germany) according to the manufacturer's instruction. Human lung tissue was homogenized in Nuclezol reagent using homogenizer (LifeReal, Hangzhou, China), while mouse lung tissue powder and hLFs can be lysed with Nuclezol reagent directly. 500 μl of Nuclezol reagent was added to each lung sample and incubated at room temperature (RT) for 5 minutes. Then 200 μl of DEPC-treated water was added to each sample, vortexed for 10 seconds, and incubated for 5 minutes. After centrifugation at 12,000 rpm for 15 minutes, the supernatant was removed to a new tube and mixed with equal volume of 100% isopropanol, followed by incubation for 10 minutes at RT and centrifugation at 12,000 rpm for 10 minutes. The supernatant was removed and the RNA precipitation was washed with 75% ethanol twice. Finally, the RNA precipitation was air dry and dissolved in RNase-free water. The concentration was measured by using Nanodrop (Thermo Scientific).

Total RNA was reverse transcribed into cDNA by using Hiffair® III Reverse Transcriptase Kit (Yeasen, Shanghai, China) according to manufacturer's protocol. The mRNA expression level of each gene was assessed by SYBR green–based qPCR

analyses. The primers were synthesized from Sangon (Shanghai, China) and the sequence was listed in Table S1. All samples were tested in triplicate by using the Hieff UNICON® qPCR SYBR Green Master Mix (Yeasen, Shanghai, China), with thermal cycling parameters of 95 °C, 10 s; 60 °C, 30 s each cycle for 40 cycles, at a thermal changing rate of 1.6 °C/s using QuantStudio5 (Applied Biosystems, Carlsbad, CA). The relative gene expression was normalized to housekeeping gene *GAPDH* (for human sample) or *18S* (for mouse sample) and calculated by the $2^{-\Delta\Delta Ct}$ method.

Histological evaluation and lung fibrosis scoring

Mouse lung tissue fixed with 4% paraformaldehyde was paraffin embedded and cut into 4 μm sections. Lung tissue sections were dewaxed in xylene, rehydrated in graded alcohols, rinsed in distilled water, and stained with hematoxylin and eosin. Collagen in mouse lung tissue was stained by using modified Masson's Trichrome staining kit (Solarbio life science, Beijing, China) following the manufacturer's instruction. The stained sections were digitally scanned by using digital pathological section scanner (PRECICE500B, Beijing, China). The extent of pulmonary fibrosis was evaluated with the modified Ashcroft score [E2]. 80-100 microscopic fields were randomly chosen under 200× magnification and a score ranging from 0 (normal) to 8 (total fibrosis) was given to each field. The mean score of all fields was calculated for each animal.

Hydroxyproline content measurement

The hydroxyproline content in mouse lung was measured by using hydroxyproline kit (Nanjing Jiancheng Bioengineering Institute, Nanjing, China) according to the

manufacturer's protocol. Briefly, lung tissue from each mouse was ground in liquid nitrogen and precisely weighed 30-100 mg of tissue into centrifuge tube with 0.5 ml of lysis buffer. Then the tubes were put into 95 °C water bath for 20 mins to allow the lung tissue completely hydrolyzed. After cooled down, the pH value was modulated to 6.0-6.8 and tissue lysate was diluted with distilled water to a total volume of 10 ml. Activated carbon was added into the tissue lysate and centrifuged to remove the color. The colorless supernatant was harvested and mixed with supplemental reagents to detect the absorbance at 550 nm. The hydroxyproline concentration of each sample could be obtained through the standard curve. The content of hydroxyproline was calculated by the following formula: Hydroxyproline content (μg/mg) = hydroxyproline concentration (μg/ml) × hydrolysate volume (10 ml) / tissue wet weight (mg).

Western Blotting

Pre-ground mouse lung tissues or hLFs were lysed in chilled RIPA lysis buffer (Beyotime, Shanghai, China) containing 1× protease and phosphatase inhibitor cocktail (Beyotime, Shanghai, China), followed with incubation on ice, centrifugation and supernatant collection. Protein concentration was determined by using BCA protein assay kit (Thermo Scientific). Equal amounts of protein were separated by 8% SDS-PAGE gel, transferred to PVDF membranes (Millipore) and blocked with 5% w/v skim milk. The membrane was incubated in the primary antibody solution against α-SMA (1:1000 dilution, Abcam), Fibronectin (1:1000 dilution, Santa cruz), type I collagen (1:1000 dilution, Abcam), type III collagen (1:1000 dilution, Abcam), LOX

(1:1000 dilution, Abcam), CTGF (1:5000 dilution, Peprotech), β-actin (1:10000 dilution, Cell Signaling Technology) or GAPDH (1:10000 dilution, Cell Signaling Technology) overnight at 4 °C and then incubated in the secondary anti-mouse or anti-rabbit IgG. The StarSignal Plus Chemiluminescent assay kit (GenStar, Beijing, China) was utilized to the membranes and the blots were developed using TANON 5200 automatic chemiluminescence imaging analysis system (Tanon, Shanghai, China). GAPDH or β-actin was used to normalize sample loading. Semi-quantitative analysis was performed on the western blot bands where intensities of the bands were measured using Image J.

Immunofluorescence Staining

hLFs were cultured on the glass coverslips and fixed with 4% paraformaldehyde for 10 min at room temperature. Then cells were washed with 1 × PBS and permeabilized with 0.1% Triton X-100 for 10 min. After washed three times with distilled water, hLFs were blocked by using 10% goat serum for 30 min, followed by incubation with primary antibody against GR (1:250 dilution, Abcam) in 1% goat serum at 4 °C overnight and fluorescent secondary antibody for 1 hour at room temperature. Cells were mounted using antifade reagent with DAPI and were observed using an Olympus IX83 fluorescence microscope under 20× objective lens. Mean fluorescence intensity of GR was measured by using Image J.

Statistics

Statistical analyses were performed by using GraphPad Prism 7 (GraphPad Software, La Jolla, CA, United States) software. The paired or unpaired t test was used to

compare the continuous variables between two groups when the data were normally distributed. The Mann-Whitney test or Wilcoxon test was used to compare the unpaired or paired variables respectively if the data were not normally distributed. For experiments with more than two groups, the data were analyzed by two-way ANOVA . Cross-correlation between CTGF and Fibronectin protein level was analyzed by using Pearson correlation test. *P* value less than 0.05 was deemed statistically significant for all analyses.

REFERENCE

E1. Raghu G, Remy-Jardin M, Myers JL, et al. Diagnosis of Idiopathic Pulmonary Fibrosis. An Official ATS/ERS/JRS/ALAT Clinical Practice Guideline. *Am J Respir Crit Care Med*. 2018;198(5):e44-e68. doi:10.1164/rccm.201807-1255ST.

E2. Hübner, R. H., Gitter, W., El Mokhtari, N. E., Mathiak, M., Both, M., Bolte, H., Freitag-Wolf, S., & Bewig, B. Standardized quantification of pulmonary fibrosis in histological samples. *BioTechniques,* 2008, 44(4), 507–517. https://doi.org/10.2144/000112729

Table S1. Primer sequence for real-time qPCR

Primer	Species	Froward sequence (5' to 3')	Reverse sequence (3' to 5')
GAPDH	Human	CAGCCTCAAGATCATCAGCA	ACAGTCTTCTGGGTGGCAGT
α-SMA	Human	GCGTGGCTATTCCTTCGTTA	ATGAAGGATGGCTGGAACAG
Fibronectin	Human	TCTCCTGCCTGGTACAGAATATGTAGTGAG	GGTCGCAGCAACAACTTCCAGGT
COL1A1	Human	TCAGAACATCACCTACCACTGC	GTCCAGAGGTGCAATGTCAAG
COL3A1	Human	GGATCAGGCCAGTGGAAATGTAAAGA	CTTGCGTGTTCGATATTCAAAGACTGTT
LOX	Human	AGGCCACAAAGCAAGTTTCTG	AACAGCCAGGACTCAATCCCT
CTGF	Human	AGGATGTGCATTCTCCAGCC	GCCACAAGCTGTCCAGTCTA
FKBP5	Human	CTGAAGGGTTAGCGGAGCAC	CATGGTAGCCACCCCAATGT
GILZ	Human	TCCTGTCTGAGCCCTGAAGAG	AGCCACTTACACCGCAGAAC
PDK4	Human	TGCCTGTACAGTTGACCCAG	CCAATGTGGCTTGGGTTTCC
PLZF	Human	TCCTGTCTGAGCCCTGAAGAG	AGCCACTTACACCGCAGAAC
SCNN1A	Human	TGCACCTGTCAGGGGAAC	GTGGATGGTGGTGTTGTTGC
GRα	Human	ACTTACACCTGGATGACCAAAT	TTCAATACTCATGGTCTTATCC
HDAC2	Human	ATGGCGTACAGTCAAGGAGG	TGCGGATTCTATGAGGCTTCA
HSD11b1	Human	CGAAATCTTGAGGTTCTCTCTGT	AAGCTCCAGGCAGTGGGATA
18S	Mouse	TTGACGGAAGGGCACCACCAG	GCACCACCACCCACGGAATCG
α-SMA	Mouse	GCTGGTGATGATGCTCCCA	GCCCATTCCAACCATTACTCC
Fibronectin	Mouse	GTGTAGCACAACTTCCAATTACGAA	GGAATTTCCGCCTCGAGTCT
Col1a1	Mouse	CCCGTTGGCAAAGATGGTAG	ACCTTGGCTACCCTGAGAAC
Col3a1	Mouse	CTGTAACATGGAAACTGGGGAAA	CCATAGCTGAACTGAAAACCACC
Lox	Mouse	CAGCCACATAGATCGCATGGT	GCCGTATCCAGGTCGGTTC
Ctgf	Mouse	AGAACTGTGTACGGAGCGTG	GTGCACCATCTTTGGCAGTG
Fkbp5	Mouse	CGGAAAGGCGAGGGATACTC	TTCCCCAACAACGAACACCA

Gilz	Mouse	GGGATGTGGTTTCCGTTAAACTGGA	TGCTCAATCTTGTTGTCTAGGGCCA
Pdk4	Mouse	GAGCTGTTCTCCCGCTACAG	CGGTCAGGCAGGATGTCAAT
PLzf	Mouse	CCCAGTTCTCAAAGGAGGATG	TTCCCACACAGCAGACAGAAG
Scnn1a	Mouse	CAAGAAGTGTGGCTGTGCTTACATCTTC	ACAGGAGGCTGACCATCGTGACAG
Tgf-β1	Mouse	GGATACCAACTATTGCTTCAGCTCC	AGGCTCCAAATATAGGGGCAGGGTC
Il1β	Mouse	GGATGATGATGATAACCTGC	CATGGAGAATATCACTTGTTGG
Il4	Mouse	CTGGATTCATCGAGAAGCTG	TTTGCATGATGCTCTTTAGG
Il6	Mouse	CTGCAAGAGACTTCCATCCAG	AGTGGTATAGACAGGTCTGTTGG
Tnf-α	Mouse	ACCCTCACACTCACAAACCA	ATAGCAAATCGGCTGACGGT
Pdgfa	Mouse	CTGGCTCGAAGTCAGATCCACA	GACTTGTCTCCAAGGCATCCTC
Pdgfb	Mouse	CACCCTCTAGCTTCGTTGC	GCCGATGGTTCGTCTTCACT
Pdgfc	Mouse	AGCATCTGGACTGGCATAGAA	GGCTGTGGATGCTCCCATTA
Edn1	Mouse	CGGGTCTTATCTCTGGCTGC	AGTTCTCCGCCGCCTTTTTA
Nr3c	Mouse	CAGTGGAAGGACAGCACAAT	TGGTATCGCCTTTGCCCATT
Hdac2	Mouse	CGGTGTTTGATGGACTCTTTG	CCTGATGCTTCTGACTTCTTG
Hsd11b1	Mouse	ACTCAGACCTCGCTGTCTCT	GCTTGCAGAGTAGGGAGCAA

SUPPLEMENTAL FIGURES

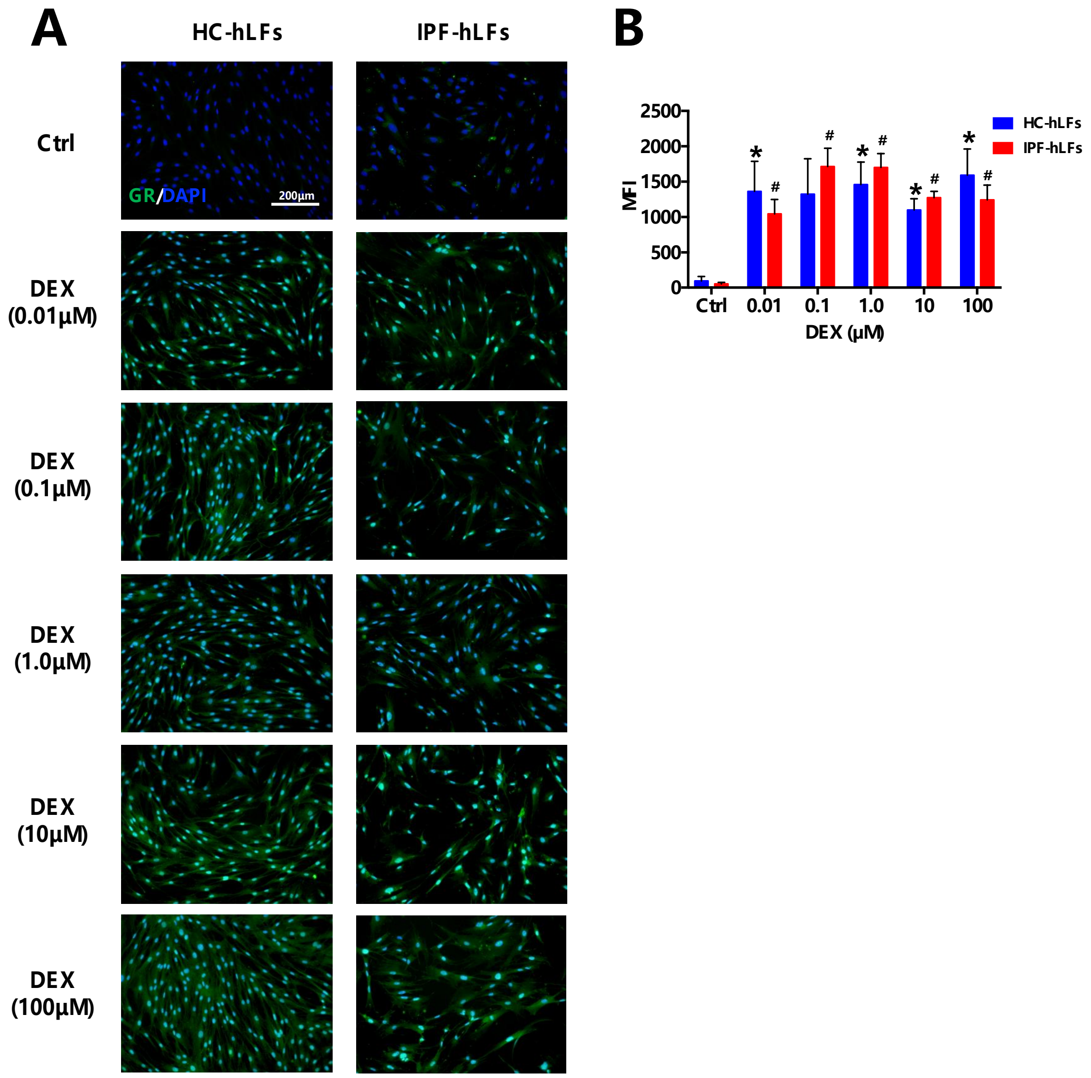

Figure S1. Dose-response relationship between the nuclear expression of glucocorticoid receptor and DEX treatment in hLFs. Human lung fibroblasts (hLFs) derived from healthy control (HC) and IPF patients were treated with DEX (0, 0.01, 0.1, 1.0, 10 and 100 μM) for 2 hours. **A.** GR was stained green and the cell nuclear was labeled by DAPI (blue). **B.** Mean fluorescence intensity (MFI) of GR in HC- and IPF-hLFs with or without DEX treatment (n = 5 and 5, respectively). Ctrl, control group, without DEX treatment. *, $P < 0.05$ when compared to HC control group. #, $P < 0.05$ when compared to IPF control group.

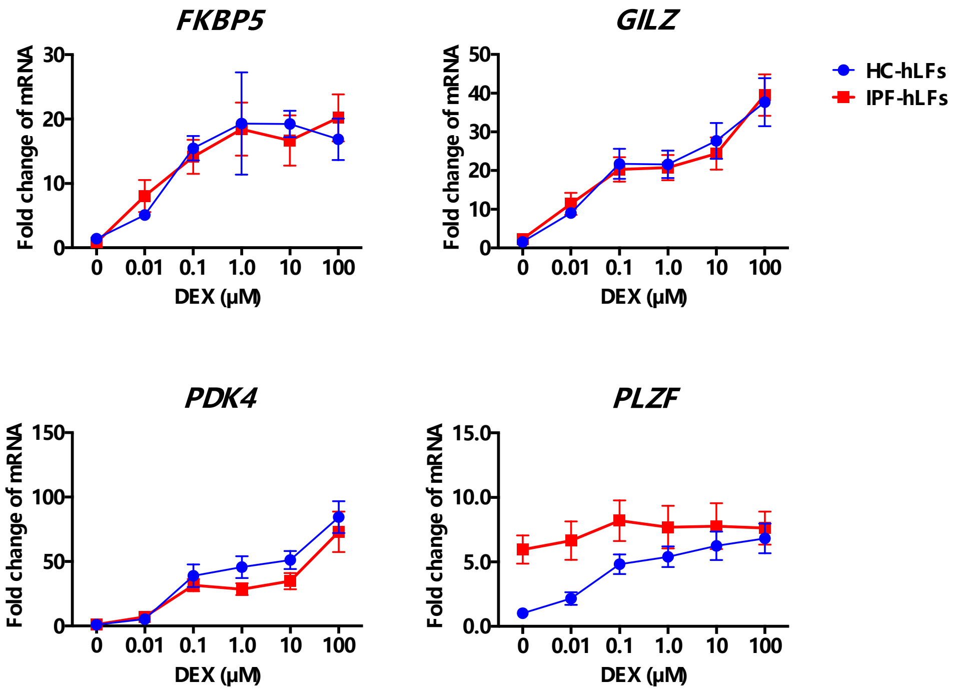

Figure S2. Dose-response relationship between the expression of glucocorticoid-inducible genes and DEX treatment in hLFs. Human lung fibroblasts (hLFs) were derived from healthy control (HC) and IPF patients (n = 6 and 7, respectively) and treated with DEX (0, 0.01, 0.1, 1.0, 10 and 100 μM) for 4 hours. Expression changes of the glucocorticoid-inducible genes were detected by RT-qPCR.

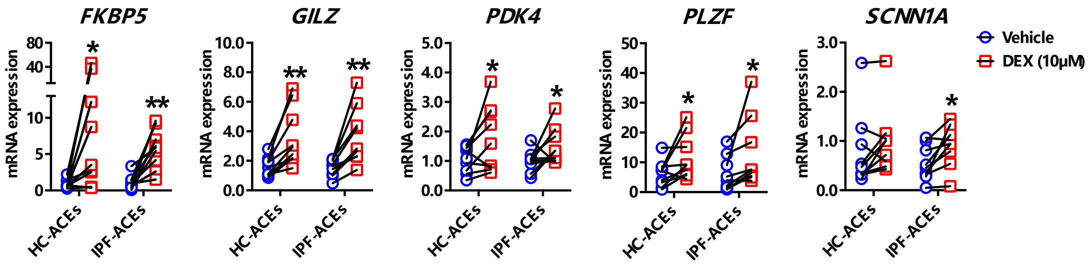

Figure S3. Expression of glucocorticoid-inducible genes in airway epithelial cells. Airway epithelial cells (AECs) were derived from healthy control (HC) and IPF patients (n = 7 and 6, respectively) and treated with 10 μM of DEX or dPBS (vehicle) for 4 hours. Expression changes of the glucocorticoid-inducible genes were detected by RT-qPCR. * and ** represents *P* value less than 0.05 and 0.01 when comparing vehicle and DEX group.

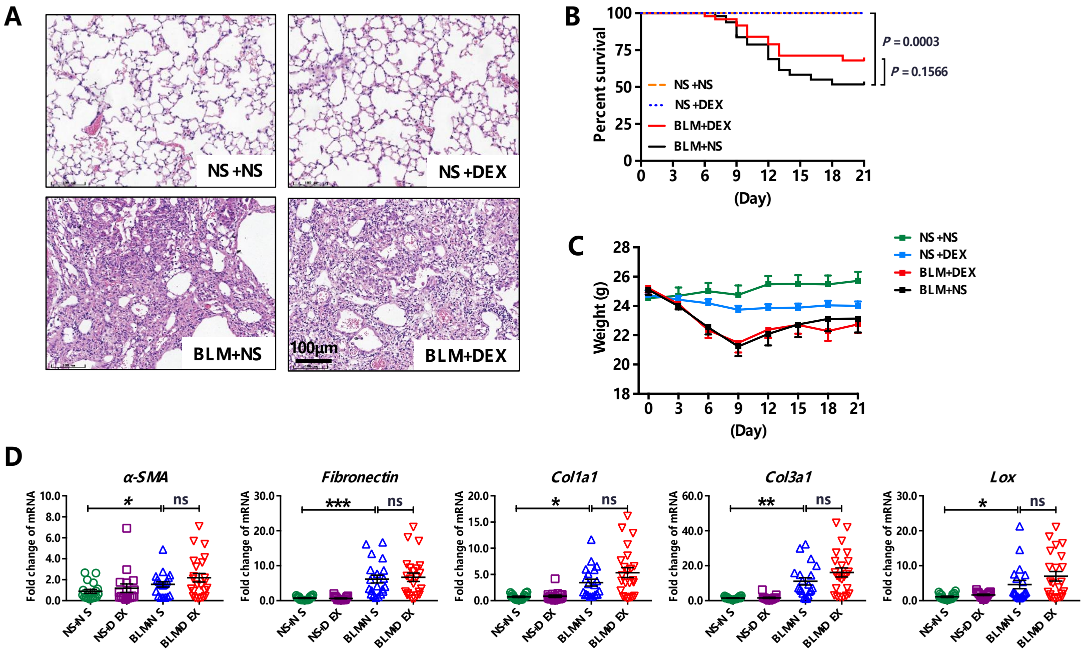

Figure S4 Effect of DEX treatment on BLM-induced lung fibrosis in mice. A. H&E staining of the mouse lung sections. “BLM/NS + DEX/NS”, mice were intratracheally administrated with bleomycin (BLM) or normal saline (NS) on the first day, and then received dexamethasone (DEX) or NS through i.p. injection once a day for the following three weeks. **B&C.** Percent survival (B) and body weight changes (C) of mice at day 0-21 after bleomycin treatment. Number of mice for each group were 26, 26, 48 and 48 initially, and 26, 26, 29 and 35 at Day 21. **D.** Transcriptional expression of the fibrosis-related factors in mouse lung tissues (n = 18, 17, 20 and 23, respectively). Data are expressed as means ± SEM. *, **, *** or ns (no significance) represents *P* value less than 0.05, 0.01, 0.001 or larger than 0.05, respectively.

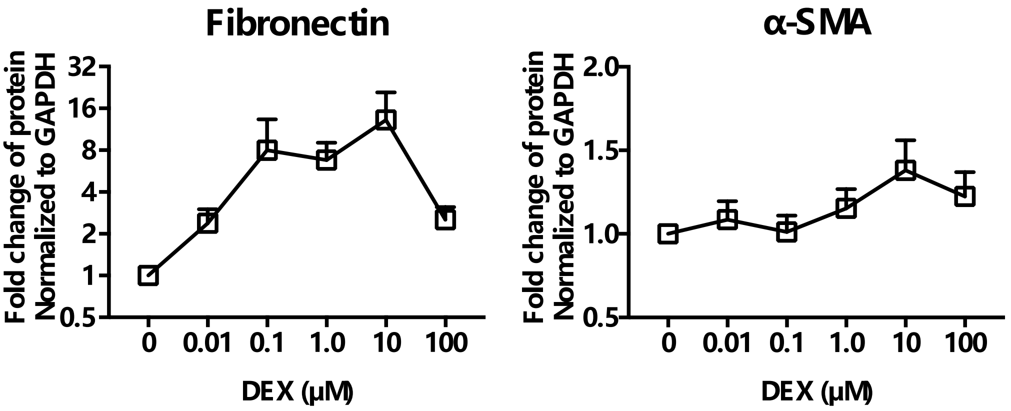

Figure S5. Expression of Fibronectin and α-SMA in hLFs upon different concentrations of DEX stimulation. hLFs derived from IPF patients were treated with different concentration of DEX (0.01, 0.1, 1.0, 10, 100 μM) for 48 hours and the protein levels of Fibronectin and α-SMA were then measured by Western blotting (n = 6).

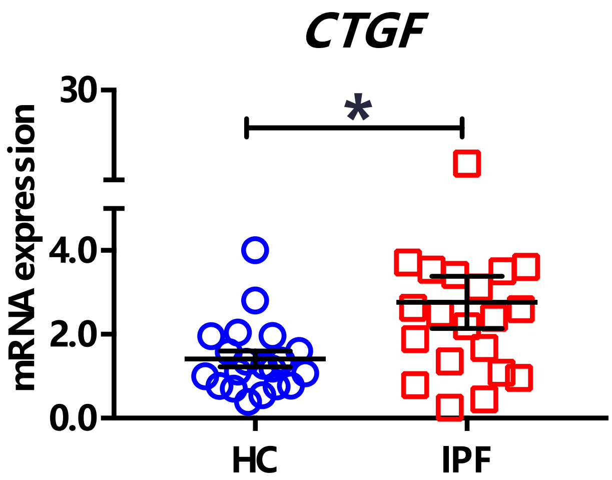

Figure S6. CTGF was upregulated in IPF lungs. Transcriptional expression of the connective tissue growth factor (CTGF) was measured in lung tissue from healthy control (HC) and IPF patients (n = 20 and 20, respectively). Data were expressed as mean ± SEM. *, *P* value < 0.05 for two-group comparison.
